# Targeting Cannabinoid CB_2_ Receptors in the Central Nervous System. Medicinal Chemistry Approaches with Focus on Neurodegenerative Disorders

**DOI:** 10.3389/fnins.2016.00406

**Published:** 2016-09-13

**Authors:** Gemma Navarro, Paula Morales, Carmen Rodríguez-Cueto, Javier Fernández-Ruiz, Nadine Jagerovic, Rafael Franco

**Affiliations:** ^1^Department of Biochemistry and Molecular Biomedicine, University of BarcelonaBarcelona, Spain; ^2^Centro de Investigación en Red sobre Enfermedades Neurodegenerativas (CIBERNED), Instituto de Salud Carlos IIIMadrid, Spain; ^3^Cell and Molecular Neuropharmacology, Institut de Biomedicina (IBUB), Universitat de BarcelonaBarcelona, Spain; ^4^Instituto de Química Médica, Consejo Superior de Investigaciones CientíficasMadrid, Spain; ^5^Center for Drug Discovery, University of North Carolina at GreensboroGreensboro, NC, USA; ^6^Departamento de Bioquímica, Facultad de Medicina, Instituto Universitario de Investigación en Neuroquímica, Universidad ComplutenseMadrid, Spain; ^7^Instituto Ramón y Cajal de Investigación SanitariaMadrid, Spain

**Keywords:** heteromer, microglia, astroglia, M0/M1/M2 phenotype, neuroprotection, neurorestoration, GPCR, amyotrophic lateral sclerosis

## Abstract

Endocannabinoids activate two types of specific G-protein-coupled receptors (GPCRs), namely cannabinoid CB_1_ and CB_2_. Contrary to the psychotropic actions of agonists of CB_1_ receptors, and serious side effects of the selective antagonists of this receptor, drugs acting on CB_2_ receptors appear as promising drugs to combat CNS diseases (Parkinson's disease, Huntington's chorea, cerebellar ataxia, amyotrohic lateral sclerosis). Differential localization of CB_2_ receptors in neural cell types and upregulation in neuroinflammation are keys to understand the therapeutic potential in *inter alia* diseases that imply progressive neurodegeneration. Medicinal chemistry approaches are now engaged to develop imaging tools to map receptors in the living human brain, to develop more efficacious agonists, and to investigate the possibility to develop allosteric modulators.

## Introduction

To date only two cannabinoid receptors have been identified and completely accepted as key members of the endocannabinoid signaling. The CB_1_ receptor (CB_1_R) is mainly expressed in the central nervous system (CNS) (Hu and Mackie, 2015), whereas, the CB_2_ receptor (CB_2_R) is mainly expressed in the periphery, especially in blood cells, and in blood-cell producing organs (Onaivi et al., 1999; Atwood and Mackie, 2010; Atwood et al., 2012). Other receptors, e.g., GPR55, the cation channel TRPV1 and the nuclear receptors of the PPAR family, are also under discussion as possible members of the endocannabinoid receptor family. CB_1_R and CB_2_R belong to the most populated family of the human proteome, i.e., to the family of receptors coupled to heterotrimeric G proteins (GPCRs). More specifically they are members of class A GPCRs, which are characterized by being structurally similar to rhodopsin, for having an extracellular N-terminal domain, a seven α-helical transmembrane domain, and a C-terminal domain of 73 (for CB_1_R) or of 59 (for CB_2_R) amino acids. Total length of the most common^1^ protein products is 472 for CB_1_R and 360 for CB_2_R. The difference in receptor length comes from the bigger N-terminal domain of the CB_1_R (116 vs. 33 amino acids).

Soon after its discovery and the realization of the relevant role of endogenous cannabinoids, the CB_1_R was considered a potential target to combat CNS diseases. In fact, the CB_1_R is considered the class A GPCR member with the highest expression in the CNS. In sharp contrast, controversy surrounds expression of CB_2_R in the CNS, and until recently this receptor was not considered as target for neurological or neuropsychiatric diseases (Atwood and Mackie, 2010; Atwood et al., 2012). This paper scans the literature that supports the view that CB_2_R may have now more potential than CB_1_R to combat some CNS disorders, in particular those related to neuroinflammatory, and neurodegenerative events. The paper also informs on current developments in medicinal chemistry aspects of CB_2_R-based CNS drug discovery.

## Better prospects for CB_2_R than for CB_1_R in CNS diseases

GPCRs constitute the target of approximately 40% of approved drugs. Drug development programs are still heavily relying on the potential of GPCRs for a huge variety of diseases. Agonists, which are able to activate the receptor and compete with the endogenous agonist, and antagonists, which block the receptor and impede activation by the endogenous agonist, have therapeutic potential. However, the number of medications that consist of GPCR antagonists outnumbers that of GPCR agonists. In general terms, the higher success of antagonists means that they have fewer side effects than agonists, although other causes overlay. The endocannabinoid system is a very special case as endogenous compounds produced by neurons and acting on central CB_1_Rs are absolutely required for higher brain functions, but any synthetic or natural (e.g., Δ^9^-tetrahydrocannabinol) agonist reaching the brain and hitting CB_1_R has proved to have psychotropic actions in animal models of disease and in humans. Therefore, the potential of CB_1_Rs as targets for diseases of the CNS, and also peripheral disorders, has been limited by the psychoactive side effects derived from their agonists, and for the need to consider the risk-benefit balance. In this context, some researchers wanted to develop CB_1_R antagonists (including inverse agonists) as a safer alternative in those pathologies having an overactivity of the endocannabinoid system (e.g., obesity, addiction, schizophrenia), although side effects were also evident with such strategy (see below).

**Figure 1:**
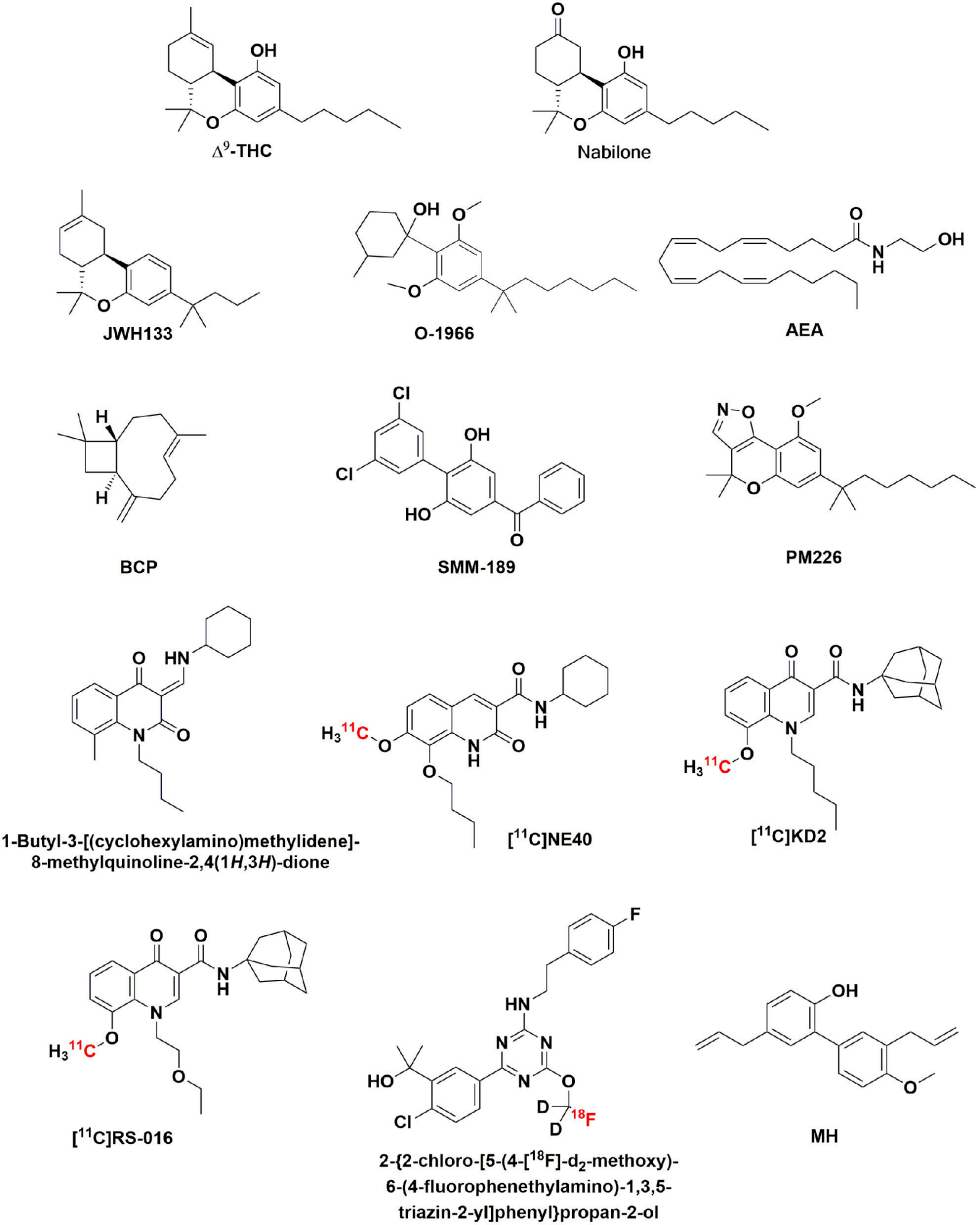
**Chemical structure of Δ^9^-THC, nabilone, and the CB_2_R ligands: JWH133, 0-1966, AEA, BCP, SMM-189, PM226, 1-butyl-3-[(cyclohexylamino)methylidene]-8-methylquinoline-2,4(1*H*,3*H*)-dione, [^11^C]NE40, [^11^C]KD2, [^11^C]RS-016, 2-{2-chloro-[5-(4-[^18^F]-d_2_-methoxy)-6-(4-fluorophenethylamino)-1,3,5-triazin-2-yl]phenyl}propan-2-ol**.

The first two molecules targeting CB_1_R that reached the therapeutic market (in the 80s) were Δ^9^-tetrahydrocannabinol, also known as dronabinol (marketed as Marinol®), and nabilone (marketed as Cesamet®) (Figure 1), both prescribed to combat nausea and vomiting, as well anorexia, derived from cancer, and AIDS treatments, respectively (Green et al., 1989), but their use was limited. By contrast, a CB_1_R antagonist/inverse agonist, rimonabant (Acomplia®), was approved in 2006 to treat obesity, and metabolic syndrome (Carai et al., 2006) and generated extremely high expectations. Unfortunately, the drug had to be retired due to side effects, especially due to reports of suicide (Sam et al., 2011). Consequently, chances, that other CB_1_R selective drug may advance though regulatory bodies, and reach the market have dramatically diminished. In this context, the CB_2_R has taken the lead in the race to find novel cannabinoid-related drugs for CNS diseases. On the one hand, CB_1_R is expressed in almost any brain region, and in many neuronal cell types, whereas CB_2_R expression in neurons is restricted to few areas. Accordingly, fewer side effects are expected when drugs are targeting receptors with restricted expression than when drugs are targeting receptors widely expressed in the CNS. Furthermore, CB_2_R are upregulated in a variety of CNS diseases that course with activated microglia or astroglia. Then the CB_2_R but not the CB_1_R is a promising candidate to consider in diseases with a neuroinflammatory component. It is even possible that the activation of CB_2_Rs may explain recent controversies in relation with the consumption of cannabis as a factor either increasing risk or preventing against spontaneous brain insults (e.g., intracerebral hemorrhage). Recent epidemiological studies suggest a potential protective effect of cannabis to the modulation of C-reactive protein response in intracerebral hemorrhage (Di Napoli et al., 2012, 2016; Alshaarawy and Anthony, 2015), an effect that could be possibly related to CB_2_R activation, although this has not been investigated. Advantages of developing CB_2_R selective drugs to prevent neurodegeneration in cases of neuroinflammation are presented later in this article.

As macrophages express CB_2_R and microglia is somehow a similar cell type, these receptors were soon identified in microglial cells, but further research demonstrated that they can be also found in other types of glial cells (see below). There is however, some controversy on the degree of CB_2_R expression in resting vs. activated microglial cells. Also the activated microglial phenotype is different in macrophages filtered from the blood into the CNS and in resident microglia that becomes activated due to, *inter alia*, accumulation of protein aggregates such as alpha-synuclein, or ß-amyloid. Remarkably, (see Franco and Fernández-Suárez, 2015 and references therein) a better understanding of the expression and role of CB_2_R in the different microglial phenotypes (M0, M1, M2) will help in designing CB_2_R selective ligands able to induce the neuroprotective/anti-inflammatory-skewed phenotype(s).

CB_2_R may be also expressed by CNS neurons. The role of CB_2_Rs in schizophrenia, depression, food consumption, and drug addiction has been demonstrated in different laboratories and the results are consistent with neuronal expression of the receptor (Onaivi et al., 2008a,b,c; Hu et al., 2009; García-Gutiérrez et al., 2010; Ishiguro et al., 2010a,b; García-Gutiérrez and Manzanares, 2011; Ortega-Alvaro et al., 2011; Aracil-Fernández et al., 2012; Navarrete et al., 2012, 2013; Bahi et al., 2014; Blanco-Calvo et al., 2014; Ortega-Álvaro et al., 2015; Rodríguez-Arias et al., 2015; García-Cabrerizo and García-Fuster, 2016). The receptor is significantly expressed in neurons in the brain stem (Van Sickle et al., 2005), in the cerebellum (Skaper et al., 1996; Ashton et al., 2006; Gong et al., 2006; Rodríguez-Cueto et al., 2014) in the internal and the external segments of the *globus pallidus* of the non-human primate (Lanciego et al., 2011), and in the *substantia nigra* (in humans, not in rodents) (García et al., 2016; Gómez-Gálvez et al., 2016). Different laboratories working with rodents or primates have also identified receptor expression in neurons of the prefrontal cortex and hippocampus (Callén et al., 2012; den Boon et al., 2012; Sierra et al., 2015; García-Cabrerizo and García-Fuster, 2016). Expression of CB_2_R in the basal ganglia show promise in Parkinson's disease and Huntington's chorea; the presence of the receptor in hippocampus and prefrontal cortex makes it attractive for Alzheimer's disease and the expression in brain stem and cerebellum opens novel therapeutic avenues for a variety of diseases such as hereditary spinocerebellar ataxias. Last but not least, the data on CB_2_R-mediated endocannabinoid regulation of microglial activation makes the receptor attractive for diseases with a neuroinflammatory component.

Cannabinoid neuroregulation is mainly based on retrograde signaling (Alger, 2002), i.e., endocannabinoids come from post-synaptic elements to activate presynaptic receptors. However, postsynaptic CB_2_Rs have been also reported (Brusco et al., 2008). The combination of restricted neuronal expression with the possibility of targeting pre- or postsynaptic receptors, makes the CB_2_R a really attractive target.

## CB_2_R in neurodegenerative disorders. relevance of differential expression of CB_2_R in neural cells

**Figure 2:**
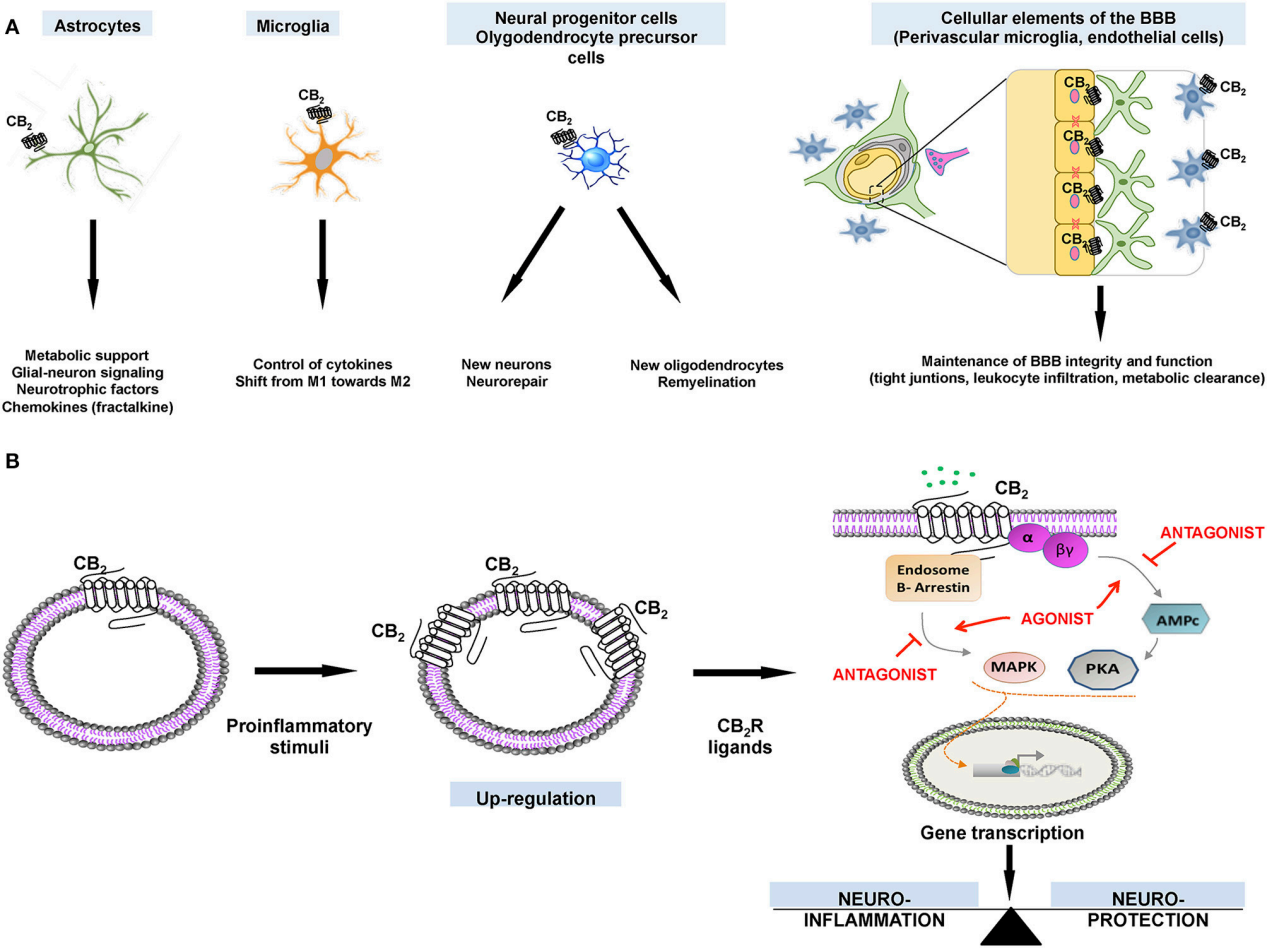
**(A)**. Expression of CB_2_Rs in different neural cell types and how receptor activation may impact on cell-specific functions. **(B)** Cellular events that explain the therapeutic possibilities for ligands that target CB_2_Rs, which are upregulated in activated glial cells.

The preservation of neuronal integrity and survival is one of the most promising therapeutic possibilities of CB_2_R-targeting cannabinoids (Atwood et al., 2012). There is potential in pain and in numerous acute or chronic neurodegenerative/neuroinflammatory conditions (Jhaveri et al., 2007; Micale et al., 2007; Campillo and Páez, 2009). The neuroprotective potential of compounds targeting the CB_2_R is, first of all, the logical consequence of their location in key cell types (e.g., in specific neuronal subsets, activated astrocytes, reactive microglia, perivascular microglia, oligodendrocytes, and neural progenitor cells), and also in some structures (e.g., the blood-brain barrier (BBB)) that are critical for the maintenance of the CNS integrity (Amenta et al., 2012; Chung et al., 2016) (Figure 2A). Such variety of locations enable compounds capable to selectively activate the CB_2_R to exert a selective control over the specific functions fulfilled by these cells in degeneration, protection and/or repair (Fernández-Ruiz et al., 2014). For example, BBB function is under the control of CB_2_R-mediated signals (Fujii et al., 2014), which maintain the integrity of tight junctions, inhibit leukocyte infiltration, and facilitate β-amyloid clearance (Vendel and de Lange, 2014).

CB_2_Rs in glial cells recruited to the site of the neurodegeneration, appear to be critical for preserving the neuronal integrity and function (Savonenko et al., 2015). In fact, CB_2_R may be absent of these cells in resting conditions, with a weak expression in the healthy brain. As the receptors are strongly up-regulated when glial cells are activated in conditions of neurodegeneration (Fernández-Ruiz et al., 2007, 2015), they have potential from a therapeutic point of view (Figure 2B). Up-regulation may occur in both astrocytes and microglial cells, but the CB_2_R-mediated signaling may vary depending *inter alia* on the type of pathology and the experimental model. CB_2_R-mediated neuroprotection/neurorestoration mechanisms are of special interest in disorders that affect movement-related areas, such as (i) Parkinson's and Huntington's diseases (affecting the basal ganglia, and producing rigidity, postural instability, bradykinesia, tremor, and chorea), (ii) autosomal dominant spinocerebellar ataxias (affecting the cerebellum and its afferent and efferent connections, and producing loss of balance, and motor incoordination), and (iii) amyotrophic lateral sclerosis (ALS) (affecting upper and lower spinal motor neurons, and producing muscle denervation and atrophy, which results in a progressive weakness and paralysis affecting voluntary muscles). For example, in this last disorder, CB_2_Rs become up-regulated in microglial cells recruited at the spinal cord of patients (Yiangou et al., 2006), a fact corroborated by studies in the TDP-43 mouse model of the disease (Espejo-Porras et al., 2015). However, apart from microglial cells, other CB_2_R-positive cells were found in this murine model (Espejo-Porras et al., 2015). In another murine model of ALS, (the SOD-1 mouse), CB_2_R also become up-regulated, but the study did not characterize the type of cell that was expressing the receptors (Shoemaker et al., 2007).

Interestingly, microglial CB_2_Rs appear up-regulated in the cerebellum of patients with different autosomal dominant cerebellar ataxias, but such trend was also found in activated astrocytes located in the cerebellar parenchyma and in the periphery of blood vessels, and in certain neuronal subpopulations (Rodríguez-Cueto et al., 2014). Similarly, increased levels of CB_2_R are found in both striatal activated astrocytes and reactive microglial cells after an insult with malonate in rats, an experimental model of Huntington's disease (Sagredo et al., 2009). Although data collected from Huntington's disease patients or obtained in genetic models of the disease (e.g., R6/1, R6/2) indicated that CB_2_R were located and up-regulated only in microglial cells (Palazuelos et al., 2009), a more recent study situated the up-regulation of these receptors in vascular cells, not in activated glial cells, in HD patients (Dowie et al., 2014).

In yet another neurodegenerative condition affecting the basal ganglia circuits, (Price et al., 2009) were the first to demonstrate up-regulation of CB_2_R in microglial cells recruited at the *substantia nigra* in MPTP-lesioned mice. In the study it was not addressed whether there were other CB_2_R-positive cells that do not correspond to reactive microglia. We investigated the issue in parkinsonian patients using *postmortem* samples and identified such up-regulation in microglial cells (labeled with Iba-1) and in another unidentified cell type (Gómez-Gálvez et al., 2016).

CB_2_R has potential in demyelinating disorders (e.g., multiple sclerosis; Molina-Holgado et al., 2002; Gomez et al., 2010, 2011). In fact, CB_2_R are present in oligodendrocytes, and more importantly, in their natural precursor cells, so that they may play a role in their survival, proliferation, and differentiation. CB_2_Rs have been also identified in neural progenitor cells, and it appears that they can play a role in the proliferation and differentiation of these precursors (Palazuelos et al., 2006, 2012; Goncalves et al., 2008; Avraham et al., 2014), opening the possibility to facilitate neurorestoration by pharmacologically manipulating this receptor. Lastly, the identification of CB_2_Rs in perivascular microglial cells in the cerebellum (Núñez et al., 2004) may be possibly related to the role attributed to these receptors at the level of the BBB (see above).

## Challenges in CB_2_R-based drug design

Pharmacology of cannabinoid receptors is complex due to the lipophilic nature of many natural and synthetic agonists. Endogenous agonists of many class A GPCRs are hydrophilic, which contrast with the lipophilic nature of endocannabinoids. Pharmacological characterization by radioligand binding to CB_2_R is especially complex. On the one hand, the binding site extends deeply within the seven transmembrane domain of the receptor, and the two available radiolabeled ligands (tritiated CP-55940 and tritiated WIN-55212-2) do not interact with exactly the same amino acid residues in the orthosteric center; in particular CP-55940 does not interact with a conserved lysine residue in the binding site (Tao et al., 1999). Furthermore, it is hypothesized that cannabinoids may not reach the binding site from the outside of the cells but by lateral diffusion via the lipid bilayer of the plasma membrane (Guo et al., 2003; Makriyannis et al., 2005; Hurst et al., 2010). These features suggest that newly synthesized drugs or newly discovered natural cannabinoids have qualitatively different modes of binding to CB_2_Rs. On the other hand, the nonspecific binding to membranes from natural CNS sources is high and leads to low-confidence values of the amount of receptor in neural cells. This problem is partially solved by performing the assays in heterologous cells expressing the human receptor; such approach provides reliable parameters for drug discovery. The complex pharmacology is also slowing the discovery of allosteric centers, and accordingly, of allosteric CB_2_R modulators.

GPCR pharmacology must somehow be revisited due to the occurrence of receptor heteromers (Cordomí et al., 2015; Franco et al., 2016). Each heteromer is unique and functionally different from the two constituting receptors. In fact, affinity of agonists/antagonists may change when a given receptor is forming heteroreceptor complexes, and more importantly, signaling cascades may be heteromer-specific (Ferré et al., 2009; Franco et al., 2016). Also relevant is the fact that presynaptic heteromers seem to be different from those in post-synaptic locations, i.e., a given GPCR may form different heteromers in pre- or post-synaptic membranes. Cannabinoid receptors may form a variety of heteromers with other class a GPCRs (see www.gpcr-hetnet.com; Borroto-Escuela et al., 2014). Interestingly, the two cannabinoid receptors may interact and give rise to CB_1_R-CB_2_R heteromers (Callén et al., 2012; Sierra et al., 2015). In agreement with the widespread distribution of CB_2_Rs in brain and the robust expression of CB_2_Rs in the *globus pallidus*, CB_1_R-CB_2_R heteromers are abundant in basal ganglia output neurons; available data indicate that these CB_1_R-CB_2_R heteromers are mainly post-synaptic. Pallidal expression of heteromers investigated in a primate model of Parkinson's disease was evident in naïve and parkinsonian animals, but it was markedly reduced in the levodopa-induced dyskinetic group (Sierra et al., 2015). Although likely, cannabinoid-receptor containing heteromers have not been identified and characterized in glial cells. Heteromer expression is worth considering on designing drugs targeting CB_2_R. In particular pallidal CB_1_R-CB_2_R heteromers constitute a specific target in Parkinson's disease. A main advantage of selectively targeting GPCR heteromers, i.e., to use drugs that preferentially act on heteromer-expressing cells, is the reduction of side effects.

## CB_2_R ligands as therapeutic agents in CNS diseases

### Positron emission tomography reagents for brain imaging

Studies of CB_2_R ligands as diagnostic agents for noninvasive brain imaging have been reported. Positron emission tomography (PET) provides a sensitive and non-invasive imaging technique to quantify CB_2_R expression in the CNS. This technique requires radioligands with high affinity and high specificity toward CB_2_R. Despite the development of highly selective CB_2_R ligands (Han et al., 2014), a limited number of PET radiotracers for imaging CB_2_R have been reported. Whereas, novel PET tracers for CB_1_R in brain imaging have been evaluated in clinical trials, few CB_2_R radioligands have been tested in humans. Few years ago, the first PET tracers for CB_2_R were presented as candidates for the *in vivo* imaging of neuroinflammatory events (Evens and Bormans, 2010). Preliminary clinical assays of the first CB_2_R radioligand, [^11^C]NE40 (Figure 1), showed appropriate fast brain kinetics in the healthy human brain (Ahmad et al., 2013). A major challenge is the development of CB_2_R PET agents with maximized brain penetration and minimized non-specific binding. In this sense, structural optimization of [^11^C]KD2 (Figure 1) (Mu et al., 2013), a potential PET tracer with poor brain penetration, led to the discovery of [^11^C]RS-016 (Figure 1), which showed slightly improved blood-brain penetration, and higher specific CB_2_R binding in murine spleen tissues and *postmortem* ALS patient spinal cord tissues (Contartese et al., 2012; Slavik et al., 2015a,b). A promising PET tracer candidate for the *in vivo* evaluation of neuroinflammation and disease progression has been recently described (Hortala et al., 2014). A triazine derivative labeled with the long-lasting radionucleotide fluorine-18 (Figure 1), 2-{2-chloro-[5-(4-[^18^F]-d2-methoxy)-6-(4-fluorophenethylamino)-1,3,5-triazin-2-yl]phenyl}propan-2-ol, showed in rhesus macaques, and baboons significant brain uptake and moderate washout.

### Current medicinal chemistry approaches

Often, increased levels of the endogenous cannabinoid, anandamide (AEA, Figure 1), correlate with neurodegenerative conditions. In recent studies, AEA has been shown to alleviate lipopolysaccharide-induced neuroinflammation in rat primary microglial cultures. Even though AEA can activate CB_1_R, CB_2_R, and other receptors such as GPR55, GPR18, TRPV1, or PPARs, the anti-inflammatory effects seem to be CB_2_R-mediated, although a possible functional cross talk with GPR18/GPR55 cannot be ruled out (Malek et al., 2015). Accordingly, AEA may have potential therapeutic action on managing microglial-derived neuroinflammation and may regulate many aspects of the brain's inflammatory response. However, from a medicinal chemistry perspective, drug development is more securely based on designing novel and selective CB_2_R ligands.

Despite the increasing number of reports on selective CB_2_R ligands and the high expectations with this cannabinoid target, only a few synthetic CB_2_R agonists have reached clinical trials (Han et al., 2014; Aghazadeh Tabrizi et al., 2016). CB_2_R agonists, namely GW842166X, CP55940, S-777469, and JTE-907, completed phase II for treatment of different pain conditions, but none of them has been evaluated in humans for neurodegenerative or neuroinflammatory diseases. However, preclinical data of CB_2_R agonists and inverse agonists have been described within this therapeutic perspective (Dhopeshwarkar and Mackie, 2014; Zhang et al., 2014).

Administration of a selective CB_2_R agonist, JWH-133 (Figure 1), to an animal model of brain infarction improved infarct outcome and neurological impairment through inhibition of different subpopulations of microglia and macrophages (Zarruk et al., 2012). Repeated treatments with the resorcinol-based CB_2_R agonist, O-1966, resulted in attenuated BBB disruption and neuronal degeneration as shown in a traumatic brain injury model (Amenta et al., 2012).

Trans-caryophyllene (BCP, Figure 1), a bicyclic sesquiterpene with selective CB_2_R agonist properties, has been reported as a therapeutic target for the treatment of cerebral ischemia (Guo et al., 2014). This sesquiterpene suppressed hypoxia-induced neuroinflammatory responses by inhibiting NF-κB activation in microglia. Effectively, studies performed in the microglial cell line BV-2 and in primary cultures of microglia indicated that the inhibitory action of both cannabinoid receptor agonists and antagonists was mediated by extracellular signal regulated kinase 1/2 (ERK1/2), cytosolic phospholipase A2 (cPLA2), and activation of nuclear factor kappa (NF-κB) (Ribeiro et al., 2013).

New potentially neuroprotective CB_2_R ligands have been recently described. Among them, the novel CB_2_R inverse agonist SMM-189 (Figure 1) (*K*_i_(CB_2_) = 121 nM; *K*_i_(CB_1_) = 4780 nM; EC_50_ = 153 nM) showed in a murine model of mild traumatic brain injury efficacy in reducing the motor, visual, and emotional deficits; such neuroprotection was seemingly achieved by modulating microglial activation (Reiner et al., 2015) and chemokine expression. Reduction of the proinflammatory markers, oetaxin, MCP-1, and IP-10 by SMM-189 suggests that SMM-189 would decrease infiltration of peripheral macrophage and other cells of the immune system implicated in neurodegeneration events (Presley et al., 2015). The chromenoisoxazole PM226 (Figure 1) has been described as a selective CB_2_R agonist (*K*_i_(CB_2_) = 13 nM; *K*_i_(CB_1_R) > 40 μM; EC_50_ = 39 nM) with neuroprotective properties *in vitro* and *in vivo* evaluations (Gómez-Cañas et al., 2016). In this study, the beneficial effects of PM226 against the toxicity caused by conditioned media generated from LPS-treated cultured BV2 cells and exposed to a striatal neuron-derived cell line in culture was shown to be mediated by CB_2_R. This neuroprotective potential was confirmed in an *in vivo* model of mitochondrial damage of striatal neurons in rats. Structure-activity relationship studies on the quinoline-2,4(1*H*,3*H*)-dione scaffold allowed the discovery of the CB_2_R agonist 1-butyl-3-[(cyclohexylamino)methylidene]-8-methylquinoline-2,4(1*H*,3*H*)-dione (Figure 1) (EC_50_(CB_2_) = 92 nM; EC_50_(CB_1_) > 10 μM) that significantly reduced the clinical symptoms of experimental autoimmune encephalomyelitis in a mouse model of multiple sclerosis (Han et al., 2015). As shown by histological analysis, oral administration of this quinoline-2,4(1*H*,3*H*)-dione(10 mg/Kg) decreased leukocyte infiltration in the spinal cord and demyelination in white matter.

New strategies involving the targeting of CB_2_R have been recently proposed for neurodegenerative and neuroinflammatory diseases. One of them has been proposed recently after reporting the mechanisms that could led to the beneficial effects of 4′-O-methylhokiol (MH, Figure 1), the major bioactive component of *Magnolia grandiflora L*., in animal models of neurodegeneration (Chicca et al., 2015). MH exerts dual actions on the endocannabinoid system by acting as CB_2_R modulator and COX-2 substrate-specific inhibitor.

Another strategy that needs to be explored is targeting CB_2_R homo o heterodimers. Homobivalent and heterobivalent ligands have been explored for several GPCRs such as opioid (Fulton et al., 2010), dopamine (Gogoi et al., 2012), or histamine receptors (Birnkammer et al., 2012). CB_1_R homobivalent and heterobivalent ligands have been designed and reported in the literature (Nimczick and Decker, 2015). In what concerns CB_2_R dimers, the first structurally bivalent compounds was designed and synthesized in 2014 (Nimczick et al., 2014). Unfortunately, these molecules have less activity and selectivity compared to their monomeric compound. Bivalent molecules showed to be weak antagonists/inverse agonists of CB_1_ and CB_2_ receptors whereas the monomeric parent was selective CB_2_R agonist (Nimczick et al., 2014). It appears that the development of bivalent drugs for CB_2_Rs is still a complex task as commented very recently (Glass et al., 2016). Reported bivalent CB_1_ receptor ligands are too short to bind both receptors simultaneously. The strategy for CB_1_ or CB_2_ receptor dimers need to be reviewed due to the fact that the ligand reaches the binding site through the lipid bilayer and the linkers are unlikely to be at the external receptor face.

Despite the promising therapeutic potential offered by CB_2_R agonists, their translational success depends on overcoming some limitations, such as immune suppression upon chronic use- or pro-inflammatory actions. There is growing evidence that CB_1_Rs are subject to ligand-biased signaling (Khajehali et al., 2015). However, ligand-biased signaling profiles of ligands at CB_2_R are still under scrutiny; certainly, upon validation, they could open new therapeutic approaches. For example, the endocannabinoid 2-arachidonoylglycerol is very potent activating the ERK1/2-MAPK pathway at low concentration, whereas the inhibition of the adenylyl cyclase and calcium pathways needs higher concentrations (Dhopeshwarkar and Mackie, 2014). In the near future allosteric modulation at CB_2_R may offer a novel therapeutic approach as allosteric modulators may both fine-tune the receptor response and minimize side-effects. Signaling-specific allosteric modulation as well as orthosteric probe dependence at CB_1_R is currently under intense focus (Morales et al., 2016). In what concerns the CB_2_R, positive and negative CB_2_R allosteric modulators still need to be discovered.

## Targeting CB_2_R in neurodegenerative disorders

As above mentioned, drugs specifically targeting CB_2_R in pallidal neurons may provide symptomatic relief in Parkinson's disease. However, neuroprotection is more likely afforded by guiding glial cells to protect or restore neuronal damage. The expression of CB_2_R by glia enables these receptors to participate in the control by glial cells of the neuronal homeostasis, integrity and survival, particularly when glial cells become reactive (Fernández-Ruiz et al., 2007, 2015). Such potential situates cannabinoid ligands acting on CB_2_Rs in a promising position for being used in neuroprotection (Figure 2B) (Fernández-Ruiz et al., 2015). Such pharmacological manipulations may be the best way to modulate the endogenous response provoked by these receptors, which are up-regulated in activated astrocytes and reactive microglia in response to inflammatory, excitotoxic and traumatic insults. Accordingly, preserving healthy neurons, or rescuing damaged neurons may be likely achieved by selecting the right agonist or allosteric modulator of CB_2_R (see Figure 2B).

In the case of activated astrocytes, the benefits derived from the activation of CB_2_R may be associated with: (i) increasing the trophic role exerted by these glial cells, including the supply of metabolic substrates to neurons (Köfalvi et al., 2016); (ii) enhancing the generation of neurotrophins (e.g., GDNF), anti-inflammatory mediators (e.g., interleukin-10, interleukin-1 receptor antagonist), and/or pro-survival factors (e.g., transforming growth factor-β) (Smith et al., 2000; Molina-Holgado et al., 2003); and (iii) inhibiting the production of chemokines (e.g., fractalkine) which contribute to neuronal damage (Sheng et al., 2009). All these effects should be likely dependent on the activation of CB_2_R, either working alone or in conjunction with CB_1_R (Stella, 2010).

Microglial cells have an added value as they are recruited to the lesion site where they become reactive and change morphology and molecular phenotype. Accordingly, CB_2_Rs are concentrated surrounding the site of action of the therapeutic drug. The benefits derived from targeting CB_2_R in activated microglia may be associated with: i) regulation of migration and proliferation at lesion sites (Walter et al., 2003; Carrier et al., 2004); (ii) regulation in the production of TNF-α and other microglia-derived neurotoxic factors (Fernández-Ruiz et al., 2007, 2015; Stella, 2010); and (iii) regulation of the balance M1 (pro-inflammatory) vs. M2 (neuroprotective) phenotypes (Mecha et al., 2013; Franco and Fernández-Suárez, 2015; Malek et al., 2015; Jia et al., 2016).

## Concluding remarks and future perspectives

The aim of this article was to collect evidence generated in the last years in support of the therapeutic potential of compounds selectively targeting the CB_2_R. We placed emphasis in the potential relevance to provoke neuroprotection/neurorestoration in neurodegenerative disorders, particularly when activation of glial elements and occurrence of local inflammatory events are involved. We have compared the advantages of targeting CB_2_Rs over targeting other elements of the endocannabinoid signaling, in particular the CB_1_Rs. Right now there are a number of advantages based on the biochemical and signaling properties of CB_2_Rs, the characteristics of the binding site, their capability to form heteromers, and very importantly, to their differential expression and function depending on the CNS region and the neural cell type. Knowledge of the exact role of CB_2_R in activated glial cells will enhance the therapeutic potential of targeting these receptors in neuroinflammatory/neurodegenerative disorders.

It would be relevant to assess which among those disorders may receive more benefit from the targeting the receptor. Also relevant are the new perspectives in the design and development of novel ligands targeting the receptor. Other issues that require additional investigation are those related to the necessary developments to translate the preclinical potential of CB_2_Rs and their ligands to the clinical scenario. This would be the major challenge in the next 5–10 years after which the first CB_2_R-based medications will, hopefully, be available. Expectations are that new formulations of selective CB_2_R ligands active at the orthosteric binding site, or acting as allosteric modulators, used alone or in combination with other licensed medicines, will be available to combat devastating neurological disorders such as Alzheimer's disease, Parkinson's disease, ataxias or amyotrophic lateral sclerosis.

## Author contributions

All authors have contributed to the writing and to design and preparation of figures. Coordination of efforts has been carried out by the senior authors (NJ, JF, RF) of the three participating laboratories.

## Funding

Open access partially supported by Grant 201413-30 from Fundació La Marató de TV3. Authors declare that personal funds are needed to carry out our research and/or to elaborate didactical materials/papers in some of our Institutions.

### Conflict of interest statement

The authors declare that the research was conducted in the absence of any commercial or financial relationships that could be construed as a potential conflict of interest.

## Footnotes

^1^Isoforms of endocannabinoid receptors have been identified (details available at www.uniprot.org.)
